# Epigenetic Silencing of *DKK3* in Medulloblastoma

**DOI:** 10.3390/ijms14047492

**Published:** 2013-04-08

**Authors:** Francesca Valdora, Barbara Banelli, Sara Stigliani, Stefan M. Pfister, Stefano Moretti, Marcel Kool, Marc Remke, Alfa H.C. Bai, Claudio Brigati, Thomas Hielscher, Massimo Romani, Tiziana Servidei, Massimo Zollo, Giuseppe Cinalli, André Oberthuer, Gian Paolo Tonini, Simona Coco

**Affiliations:** 1 Department of Experimental Medicine (DIMES), University of Genoa-IRCCS A.O.U. San Martino–IST National Cancer Research Institute, Genoa 16132, Italy; E-Mail: francescavaldora@libero.it; 2 Laboratory of Tumor Genetics and Epigenetics, IRCCS A.O.U. San Martino–IST National Cancer Research Institute, Genoa 16132, Italy; E-Mails: barbara.banelli@istge.it (B.B.); claudio.brigati@istge.it (C.B.); massimo.romani@istge.it (M.R.); 3 Center of Physiopathology of Human Reproduction, Obstetrics and Gynecology Unit, IRCCS A.O.U. San Martino–IST National Cancer Research Institute, Genoa 16132, Italy; E-Mail: sara.stigliani@hsanmartino.it; 4 Division of Pediatric Neurooncology, German Cancer Research Center (DKFZ), Heidelberg 69120, Germany; E-Mails: s.pfister@dkfz.de (S.M.P.); m.kool@dkfz.de (M.K.); marcremke@gmail.com (M.R.); 5 Department of Pediatric Oncology, Hematology & Immunology, Heidelberg University Hospital, Heidelberg 69120, Germany; 6 CNRS-LAMSADE Laboratoire d’Analyse et Modélisation de Systèmes pour l’Aide à la decision Paris 75775, France; E-Mail: stefano.moretti@dauphine.fr; 7 Université Paris-Dauphine, Paris 75775, France; 8 Developmental & Stem Cell Biology Program, the Hospital for Sick Children, Toronto M5G 1X8, Canada; 9 Institute of Digestive Disease, Faculty of Medicine, The Chinese University of Hong Kong, Hong Kong; E-Mail: alfa_bai@hotmail.com; 10 Division of Biostatistics German Cancer Research Center (DKFZ), Heidelberg 69120, Germany; E-Mail: t.hielscher@dkfz.de; 11 Division of Pediatric Oncology, Catholic University, Rome 00198, Italy; E-Mail: tservidei@rm.unicatt.it; 12 CEINGE, Centro di Ingegneria Genetica e Biotecnologia Avanzate, Naples 80145, Italy; E-Mail: zollo@ceinge.unina.it; 13 Pediatric Neurosurgery, Santobono-Pausilipon Children’s Hospital, Naples 80122, Italy; E-Mail: giuseppe.cinalli@gmail.com; 14 Department of Pediatric Oncology and Hematology, University Children’s Hospital of Cologne, Cologne 50924, Germany; E-Mail: andre.oberthuer@uk-koeln.de; 15 Laboratory of Neuroblastoma, Onco/Hematology Laboratory Department SDB University of Padua, Pediatric Research Institute, Padua 35127, Italy; 16 Lung Cancer Unit IRCSS San Martino Hospital-IST National Cancer Research Institute, Genoa 16132, Italy; E-Mail: simona.coco@hsanmartino.it

**Keywords:** medulloblastoma, Wnt antagonists, DKK family, DKK3 downregulation, histone deacetylase, TSA

## Abstract

Medulloblastoma (MB) is a malignant pediatric brain tumor arising in the cerebellum consisting of four distinct subgroups: WNT, SHH, Group 3 and Group 4, which exhibit different molecular phenotypes. We studied the expression of Dickkopf (DKK) 1–4 family genes, inhibitors of the Wnt signaling cascade, in MB by screening 355 expression profiles derived from four independent datasets. Upregulation of *DKK1*, *DKK2* and *DKK4* mRNA was observed in the WNT subgroup, whereas *DKK3* was downregulated in 80% MBs across subgroups with respect to the normal cerebellum (*p* < 0.001). Since copy number aberrations targeting the *DKK3* locus (11p15.3) are rare events, we hypothesized that epigenetic factors could play a role in DKK3 regulation. Accordingly, we studied 77 miRNAs predicting to repress *DKK3*; however, no significant inverse correlation between miRNA/mRNA expression was observed. Moreover, the low methylation levels in the *DKK3* promoters (median: 3%, 5% and 5% for promoter 1, 2 and 3, respectively) excluded the downregulation of gene expression by methylation. On the other hand, the treatment of MB cells with Trichostatin A (TSA), a potent inhibitor of histone deacetylases (HDAC), was able to restore both *DKK3* mRNA and protein. In conclusion, *DKK3* downregulation across all MB subgroups may be due to epigenetic mechanisms, in particular, through chromatin condensation.

## 1. Introduction

Medulloblastoma (MB) is a highly malignant embryonic tumor of the cerebellum and accounts for 20% of all intracranial tumors of childhood [1]. Although MB has been considered a unique disease, several studies demonstrated remarkable intertumor heterogeneity, suggesting distinct molecular subgroups [2–5]. Current patient risk stratification is based on clinical factors (age at diagnosis, metastatic disease and extent of resection), as well as histological subgroups (classic, desmoplastic, large cell anaplastic MB) [6]. The current consensus is that there are four major subgroups, named WNT, SHH, Group 3 and Group 4 [7,8].

Deregulation of Wingless (Wnt), Sonic Hedgehog (SHH) and Notch signaling pathways play a critical role in MB pathogenesis [9]. Two main classes of Wnt signaling antagonists have been discovered; both prevent the ligand-receptor interaction. The first class binds Wnt proteins and includes the secreted Frizzled-related protein (sFRP) family, Wnt inhibitory factor-1 (WIF-1) and Cerberus. The second class comprises members of DKK family, which bind one subunit of the Wnt receptor complex [10,11]. Members of DKK and sFRP gene families have been reported to act as tumor suppressor genes in several malignancies [12–16]. The epigenetic downregulation of *DKK1* in MB tumors and cell lines was first described by Vibhakar *et al.*[17]. In this tumor, inhibitors of histone deacetylases (HDAC), such as Trichostatin A (TSA), have been reported to induce re-expression of several genes, including *CASP8* and *DKK1*[17–19].

However, a comprehensive study on DKK family gene regulation in MB has not been published yet. In the present study, we show for the first time that *DKK3* gene is significantly downregulated in all MB subgroups as compared to normal cerebellum, whereas *DKK1*, *DKK2* and *DKK4* are overexpressed in WNT tumors. Moreover, we investigated the mechanisms of *DKK3* regulation and found that epigenetics is a key regulator of this gene.

## 2. Results and Discussion

**Figure 1:**
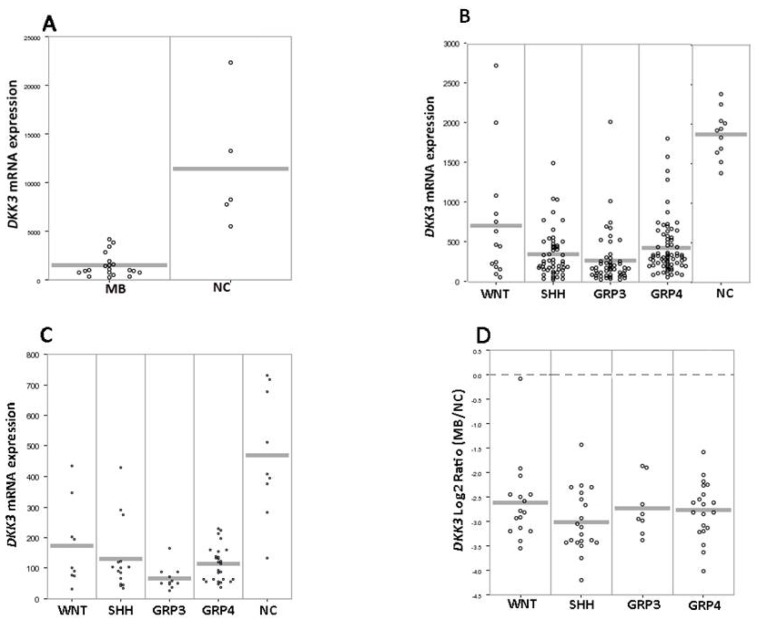
*DKK3* expression in normal cerebellum (NC) and medulloblastoma (MB) samples. Dot plots of *DKK3* expression values from expression profiles in four independent datasets: (**A**) 19 MB tumors and two pools of NC (present dataset); (**B**) 188 MBs and 11 NC data [4]; (**C**) 62 MB data [2] and nine NC [20]; (**D**) 64 MB data [5]. The statistically significance was calculated by one-way analysis of variance (ANOVA) between MB samples and NC; the differences between four subgroups were calculated by two-tailed Student’s *t*-test. *p*-values <0.01 were considered to be statistically significant. The X-axis indicates the four molecular subgroups according to the current consensus ([7]; WNT, SHH, Group 3 (GRP3) and Group 4 (GRP4)); the dataset A was not divided in the subgroups, due to a low number of samples. Y-axis: *DKK3* mRNA expression value in the (**A**), (**B)** and (**C**) dataset, respectively. The dataset (**D**) reports the relative expression between MB samples and NC, and the dotted grey line delineates the expression level in a pool of NC samples. Abbreviations: NC, normal cerebellum; MB, medulloblastoma; GRP3, Group 3; GRP4, Group 4.

We evaluated the expression of DKK family members (*DKK1*, *DKK2*, *DKK3* and *DKK4*), by screening 355 expression profiling data, including 333 MB tumors and 22 normal cerebella from four independent datasets. This analysis showed the significant *DKK1, DKK2* and *DKK4* upregulation (*p* < 0.01) in WNT subgroup tumors (Figures S1–S3), whereas *DKK1*, *DKK2* and *DKK4* were either not expressed or expressed at only very low levels in non-WNT tumors or normal cerebellum. Only a subset of SHH tumors shows some *DKK2* expression. Our findings are in agreement with Northcott *et al.*[3], who showed the overexpression of *DKK1* mRNA and protein in the WNT subgroup. The WNT-specific overexpression of *DKK1*, *DKK2* and *DKK4* suggests the activation of a negative feedback loop in this peculiar subgroup of tumors. On the contrary, we found that *DKK3* gene expression was significantly (*p* < 0.001) downregulated in all subgroups of MB compared to normal cerebellum (Figure 1).

**Figure 2:**
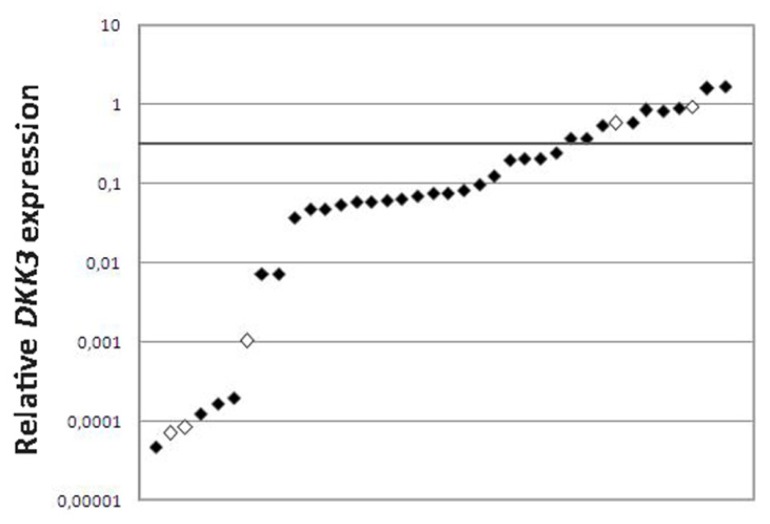
*DKK3* expression in MB samples and cell lines. Scatter plot of *DKK3* relative expression values in 33 MB samples (black diamonds) and five MB cell lines (white diamonds) by qPCR. *DKK3* relative expression compared to a pool of 10 NCs from children (age range 0–16 years). Cut-off was set at 0.5 (black line), MBs with *DKK3* expression value lower than this value was defined as downregulated. *DKK3* expression values are transformed in logarithmic scale. Y-axis: relative *DKK3* mRNA expression. Abbreviations: NC, normal cerebellum; MB, medulloblastoma.

To confirm the downregulation of *DKK3* gene expression, we validated the expression results in 33 MB tumors and five human MB cell lines by quantitative Polymerase Chain Reaction (qPCR) compared to a pool of normal cerebella and found that *DKK3* expression was reduced in 27/33 (82%) MBs and in 3/5 (60%) cell lines (Figure 2).

We then explored different genetic and epigenetic mechanisms, alone or in cooperation between themselves, that could explain the *DKK3* downregulation. First, we investigated if the somatic copy number changes, as well as the 11p loss and the focal event affecting the *DKK3* locus may contribute to drive its down-modulation. We analyzed both the status of chromosome 11 in 77 MBs (17 from dataset A and 60 from dataset D) and the focal aberration targeting *DKK3* by SNPs study [21] in 1,087 MBs. We found that 11 monosomy (10/77) and structural 11p loss (3/77) copy number aberrations were rare events and, in addition to the total absence to focal aberrations targeting *DKK3*, suggest that other regulatory mechanisms may exist.

Deregulation of miRNAs have been found in several cancers; thus, miRNAs may play a role in gene expression modulation. Therefore, we studied the miRNAs as a possible mechanism of *DKK3* epigenetic silencing. We interrogated five miRNA target prediction programs—miRWalk, Diana-microT, miRanda, miRDB and TargetScan—and selected 147 miRNAs found in at least three out of five predictive tools. Hence, we investigated the inverse correlation integrating data between miRNAs and *DKK3* expression by *in silico* analysis on the available 25 MB tumors from dataset D, containing 77 out of 147 predicted miRNAs. However, the respective Pearson’s rank correlation coefficient did not show any significant inverse correlation (*r*^2^ ≤ −0.49) with *DKK3* transcript levels (Table S1). This result is in contrast with Haug *et al.*[22], who have identified miRNA-92 as the main modulator of *DKK3* expression in neuroblastoma. The absence of modulation mediated by miRNA in MB with respect to the previous study on neuroblastoma may be explained by the activation of miRNA-92 by *MYCN. MYCN* is an oncogene frequently amplified in neuroblastoma, while its amplification is a rare event in MB (about 5% of the tumors) [23] and more frequently associated with SHH and Group 4 MBs [21].

It has been reported that chromatin remodeling by DNA methylation and histone acetylation, represents an important mechanism of inactivation of tumor suppressor genes in several cancers, including MB [17,24,25]. In this respect, *DKK3* has been observed to be methylated in several tumors [26,27], and the combined action of the HDAC inhibitor TSA and of the DNA methyltransferase (DNMT) inhibitor 5-aza-2′-deoxycytidine reactivates *DKK3* expression in breast cancer cells [28]. To determine the role of epigenetic mechanisms in regulating the expression of *DKK3* in MB, we initially performed an absolute quantitative methylation analysis on the three *DKK3* promoters in MB cell lines and primary tumors. Our results show low levels of methylation (promoter 1: 3.13%, promoter 2: 5.41%, promoter 3: 4.88%; Table S2; for representative pyrograms, see Figure S4), indicating that DNA methylation likely is not involved in *DKK3* downregulation.

**Figure 3:**
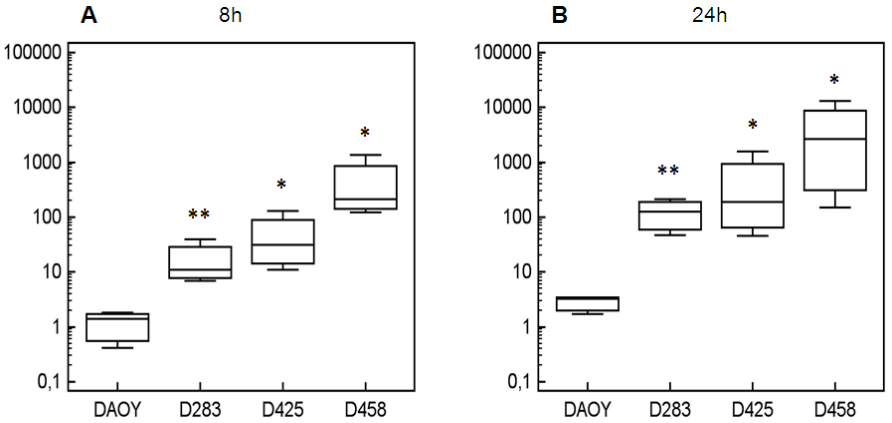
*DKK3* modulation expression after Trichostatin A (TSA) treatment in four MB cell lines. Box plots display the *DKK3* relative expression in four MB cell lines—DAOY, D283Med, D425Med and D458Med—after treatment with 20 nM of TSA at 8 h (**A**) and 24 h (**B**) with respect to untreated cells (control cells). Every experiment was performed in triplicate. Independent sample *t*-test was applied to compare ΔCt of TSA treated cells *versus* ΔCt control cells; *p*-values <0.05 were considered significant (* *p*-value <0.05; ** *p*-value <0.01). Y-axis: relative *DKK3* expression values with respect to control cells.

As a further attempt to understand if other epigenetic mechanisms beside DNA methylation could affect *DKK3* expression, we treated human MB cell lines, D425Med and D458Med, with the HDAC inhibitor, TSA. In addition, two MB cell lines, DAOY and D283Med, with *DKK3* expression comparable to normal cerebellum, were also treated with TSA. Interestingly, the TSA treatment induced a significant increase of *DKK3* expression relative to untreated cells in three out of four cell lines (D283Med; D425Med; D458Med) (Figure 3). *DKK3* expression was measured after 8 h and 24 h and increased on average 19- and 130-fold in D283Med, 57- and 602-fold in D425Med and 563- and 5296-fold in D458Med. DAOY, with *DKK3* expression levels comparable to normal cerebellum, showing no significant increase of *DKK3* expression after TSA treatment.

**Figure 4:**
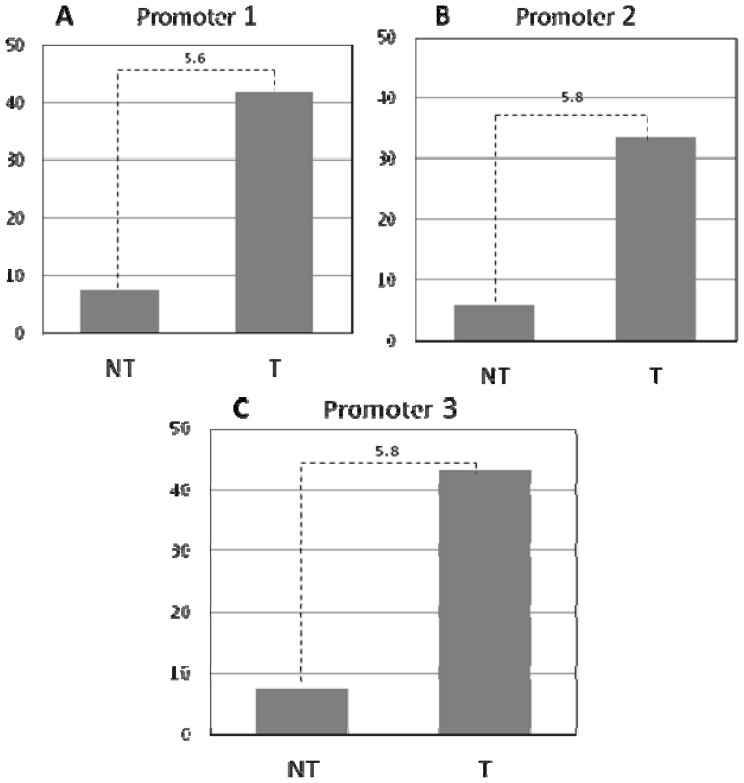
Chromatin Immuno-Precipitation (ChIP) analysis. Analyses of histone H3K4me2 levels in the three promoters of *DKK3* gene—promoter 1, promoter 2 and promoter 3, respectively—by ChIP and subsequent qPCR. The experiments were performed on D458Med cell line comparing the fold enrichment over background after 24 h of TSA treatment (T) with respect to untreated cells (UT). Signals of target DNA obtained from UT cells served as the base and were defined as one. Fold change with respect to irrelevant IgG indicates 2^−ΔΔCt^ of target DNA in treated cells over 2^−ΔΔCt^ of target DNA in UT cells (Y-axis).

In order to establish the role of histone modifications as an epigenetic regulator for *DKK3* in MB, we performed Chromatin Immuno-Precipitation (ChIP) targeting the three promoter regions of *DKK3* using antibodies against the active chromatin histone marker, H3K4me2. This experiment was conducted using D458Med cells, due to the strongest *DKK3* upregulation upon TSA treatment. Consistent with our previous results, after 24 h of TSA treatment, H3K4me2 increased about six-fold in each *DKK3* promoters with respect to the untreated cells (Figure 4).

**Figure 5:**
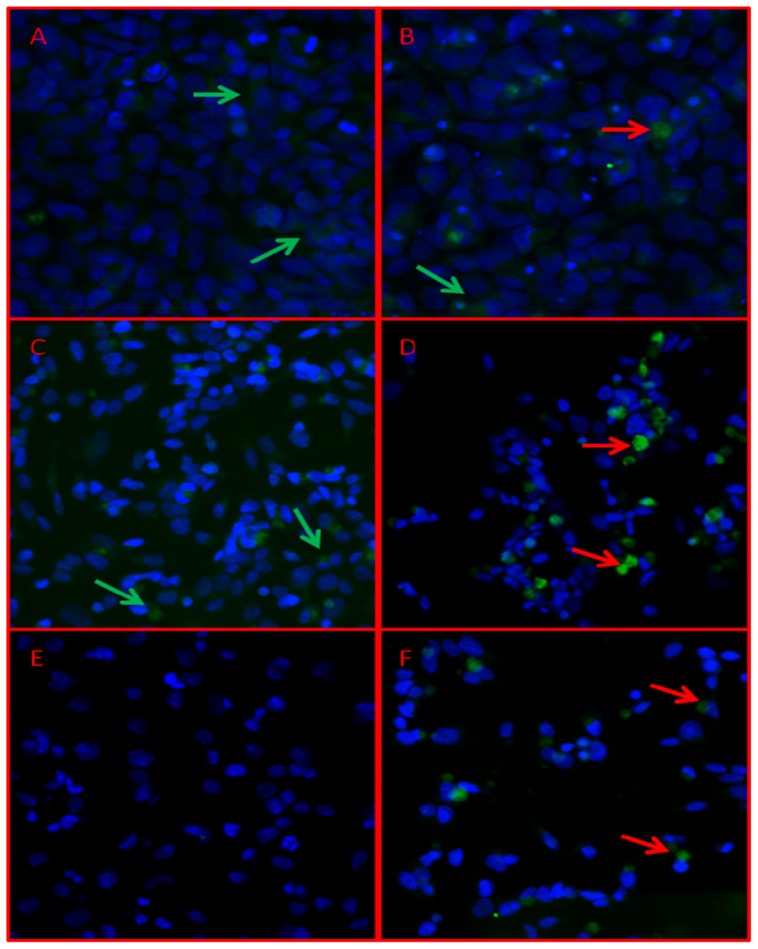
Immunofluorescence analysis of Dkk3 protein expression. Dkk3 protein expression in three MB cell lines: DAOY, D283Med and D425Med, before (**A**, **C**, **E**) and after (**B**, **D**, **F**) TSA treatment by immunofluorescence. Dkk3 positive signals (green) are evident in the cytoplasm of some cells before (**A**, **C**) and after 24 h of TSA treatment (**B**, **D**, **E**). D425Med, epigenetically silenced, after treatment, showed positive signals in about 20% of cells. The nucleus was stained in DAPI. Images were captured by Image capture system AxioVision Release 4.6 (Magnification 40X). Green arrows indicate an example of weak Dkk3 expression, while red arrows a high Dkk3 expression.

Finally, in order to evaluate if the effect of the HDAC inhibition acts also on protein expression, we evaluated the Dkk3 by immunofluorescence staining of three adherent MB cell lines after 24 h of TSA treatment (Figure 5). In agreement with the mRNA expression data, TSA treatment did not modify Dkk3 protein expression in DAOY cells. About 10% of cells had a weak positive signal compared to 8% in control cells, while the treatment in D283Med increased the Dkk3 protein from 7% to 26% (*p* = 0.060). The immunofluorescence signal in the D425Med, whose *DKK3* transcript and protein were undetectable in control cells, revealing a strong positive expression in about 20% of cells after TSA treatment (*p* = 0.025). The increase of Dkk3 protein expression was approximately 20% in both cell lines upon only 24 h at 20 nM of TSA, a sub-lethal dose.

HDAC inhibitors, like TSA, can induce a relaxed chromatin conformation independently from promoter methylation [29,30]. Our results indicate that *DKK3* reactivation in MB is accompanied by the increase of H3K4me2, a histone marker characteristic of transcriptionally active chromatin remodeling. It thus appears that the *DKK3* gene in MB is in a partially active conformation and that HDAC inhibitors can induce re-expression of *DKK3*.

## 3. Experimental Section

### 3.1. Tumor Samples and Cell Lines and Acid Nucleic Isolation

MB tumor samples were obtained from 55 pediatric patients at time of diagnosis, prior to any radio- or chemo-therapy (Table S3). Written informed consent was obtained from all the patients’ parents or legal guardians. Approval from the Ethical Committee was obtained (July 11, 2011). Ten samples of normal cerebella from children (6 Caucasians and 4 African Americans; ages: 0–16 years, cause of death: sudden death provided by NICHD Brain and Tissue Bank, Baltimore, MD, USA) and a commercial pool (Clontech, Cambridge, UK) from female and male Caucasians (ages: 16–70 years; cause of death: sudden death) were used as reference samples. The human MB cell lines—DAOY, D341Med and D283Med—were from the American Type Culture Collection and D425Med, D458Med cells were kindly provided by Prof. G. Basso, Padua, Italy. Genomic DNA was isolated from about 50 mg of snap-frozen tumor tissue or from 2 × 10^6^ cells using the standard phenol-chloroform protocol. DNA concentration and purity were evaluated by Biophotometer (Eppendorf; Hamburg, Germany). Total RNA was extracted from tumors and cells using the miRNeasy Mini kit (Qiagen; Hilden, Germany); RNA quality control and quantification were carried out by the 2100 Bioanalyzer instrument using RNA 6000 Nano kit (Agilent Technologies; Santa Clara, CA, USA). Only RNA samples with a RIN (RNA Integrity Number) of at least 7 were included in the study.

### 3.2. Gene Expression Profiling, Array-CGH Data and miRNA

Gene expression profiling was performed on 19 MB samples and two pools of normal cerebellum, as reference samples using the 44 k whole genome oligonucleotides microarray (Agilent Technologies; Santa Clara, CA, USA) (Dataset A). Labeling and hybridization of samples was performed according to the “one-color microarray-based gene expression analysis” protocol. The reference samples, including a pool of normal cerebellum from children and a commercial pool from adults, were hybridized in triplicate and duplicate, respectively. The data were extracted using Feature Extraction software (v. 9.5; Agilent Technologies; Santa Clara, CA, USA, 2009); probe intensities were log base 2-transformed and normalized across arrays with the scale normalization method implemented in R package “limma” version 3.10.2. All raw data were deposited in Gene Expression Omnibus (http://www.ncbi.nlm.nih.gov/geo/; GSE39182). We also included the data obtained from three independent public datasets of gene expression: (1) B, including 188 MBs and 11 normal cerebellum data [4]; (2) C, including 62 MB data [2] and 9 normal cerebellum [20]; (3) D, including 64 MB data [5]. Data are accessible through the open access database R2 for visualization and analysis of microarray data (http://r2.amc.nl). Differences between the comparisons of MBs *versus* normal cerebellum were studied using the one-way analysis of variance (ANOVA), while differences between four subgroups were calculated by two-tailed Student’s *t*-test; *p* values < 0.01 were considered to be statistically significant. Array-CGH data were available for 77 MBs already published by Coco *et al.*[31] (17 MBs, 244 k Agilent Technologies; GEO accession number GSE23005) and Pfister *et al.*[32] (60 MBs, 6 k BAC array; GEO accession number GSE8634). The raw data were analyzed by Agilent Genomics Workbench Lite Edition software (v. 6.5; Agilent Technologies, Santa Clara, CA, USA, 2010) using the z-score algorithm. SNPs data of 1,087 MBs were by Nothcott *et al.*[21]. The microRNAs targeting *DKK3* was determined interrogating five target prediction programs: miRWalk, Diana-microT (v. 3.0); miRanda (August 2010); miRDB (April 2009); and TargetScan (v. 5.1). Statistical analysis was performed calculating the level of inverse correlation between miRNA/mRNA combined miRNAs and *DKK3* expression values from 25 tumors from dataset D by respective Pearson’s rank correlation coefficient; −1 < *r*^2^ > 1; and a significant negative correlation was defined as *r*^2^ ≤ −0.5.

### 3.3. qPCR Analysis

Total RNA (50 ng) was amplified and reverse transcribed using the WT-Ovation RNA Amplification System (NuGEN Technologies; San Carlos, CA, USA), according to the manufacturer’s instructions. The expression value of *DKK3* was evaluated in 33 MBs and 5 MB cell lines by qPCR using specific Taqman gene expression assays (Hs00951307_m1; specific for 6–8 exons, Applied Biosystems, Carlsbad, CA, USA). *EIF4A2* and *ATP5B* were used as reference genes (Primer Design, Southampton, UK). All reactions were performed in duplicate on the Mastercycler® RealPlex^4^ System (Eppendorf), and the downregulation relative to the pool of normal cerebellum was defined for values lower than 0.5 using the 2^−ΔΔCt^ formula.

### 3.4. Trichostatin a Treatment and Chromatin Immuno-Precipitation (ChIP) Assay

DAOY, D283Med, D425Med and D458Med MB cell lines were treated with 20 nM Trichostatin A (TSA) (Upstate Biotechnology, Lake Placid, NY, USA) for 8 h and 24 h. Untreated control cells were included in each experiment. The final concentration of TSA was chosen according to Vibhakar *et al.*[17]. For qPCR expression analysis, total RNA was extracted from MB cells after 8 h and 24 h of TSA treatment. ChIP assays for the Histone 3 K4 dimethylated (H3K4me2) were performed on D458Med after 24 h of TSA treatment using the EpiQuik Methyl-Histone H3-K4 ChIP kit (Epigentek, NY, USA), as previously described by Brigati *et al*. [30]. Every ChIP for H3K4me2 was coupled to ChIP reactions with the irrelevant normal mouse IgG. Specific primers to amplify the three promoter regions of *DKK3* were designed and qPCR conditions optimized (Table S4). ChIP-qPCR data were normalized according to the Fold Enrichment Method (2^−ΔΔCt^) with respect to the irrelevant IgG.

### 3.5. Methylation Analysis

The methylation status of the three promoters of *DKK3* gene was performed on 32 tumor samples MB tumors and five cell lines by pyrosequencing analysis. Briefly, genomic DNA (1 μg) was modified with sodium bisulfite, which converts the unmethylated C into U, using the EZ DNA Methylation Gold kit (Zymo Research, Irvine, CA, USA), according to the manufacturer’s recommendations. The pyrosequencing analysis was carried out as described by Banelli *et al.*[33] with a SPQ 96MA instrument (Qiagen, Hilden, Germany) and conducted with the Pyro Q-CpG software (version 1.0.9; Qiagen Technologies, Hilden, Germany, 2006). The primers were designed with the Pyrosequencing Assay Design Software (Qiagen Technologies, Hilden, Germany). The sequence of the primers used pyrosequencing and the PCR conditions are indicated in the Table S4.

### 3.6. Immunofluorescence Analysis

Immunofluorescence analysis was performed on adherent cell lines (DAOY, D425Med and D283Med). Cells were treated for 24 h with 20 nM TSA, then control and treated cells were plated overnight on a Poly-d-Lysine 8-well slide (BD Biosciences, Franklin Lakes, NJ, USA) to allow adherence to the slide surface. Immunostaining was performed, as previously described by Del Grosso *et al*. [34], with slight modifications. Briefly, the primary antibody goat anti-human DKK-3 (H-130; Santa Cruz Biotechnologies, Inc., Santa Cruz, CA, USA) was incubated for 2 h at 37 °C, and after the secondary antibody incubation, cells were washed and stained with DAPI (1:5000). The images were acquired using an Axio Imager M1 microscope equipped with fluorescence lamp (Zeiss Inc., Oberkochen, Germany).

## 4. Conclusions

In conclusion, we describe for the first time the downregulation of *DKK3*, a Wnt pathway inhibitor, across all MB subgroups, suggesting a generally important role in MB tumorigenesis. We also demonstrate the modulation of *DKK3* at transcript and protein levels by TSA, a potent inhibitor of HDAC, in the absence of promoter methylation and miRNAs regulation. We report the specific upregulation of the other DKK members, *DKK1, DKK2* and *DKK4*, suggesting a negative feedback loop within WNT tumors. This paper demonstrates that *DKK3* is an epigenetically regulated gene, and further studies are needed to establish the role of *DKK3* and the effect of its overexpression in medulloblastoma.

## Supplementary Information
