# Supplementary material for: Epigenetic Silencing of DKK3 in Medulloblastoma
Source: Int J Mol Sci. 2013 Apr 8;14(4):7492–505. doi: 10.3390/ijms14047492 (PMC3645699; doi:10.3390/ijms14047492)
Supplement: Supplementary file 1 [file ijms-14-07492-s001.pdf]

Supplementary Information

Figure S1. Dot plots of *DKK1* expression values in medulloblastoma samples (MB) and normal cerebellum (NC) from four independent datasets by gene expression analysis: (**A**) 19 MB tumors and two pools of NC (present dataset); (**B**) 188 MBs and 11 NC data [4]; (**C**) 62 MB data [2] and nine NC [20]; (**D**) 64 MB data [5]. Differences between two groups were studied using the two-tailed Student's *t*-test (MB samples versus NC and WNT subgroup *versus* NC in dataset **A**, **B**, **C**, respectively). *p* values < 0.01 were considered to be statistically significant (**). The X-axis indicates the samples grouped into the four molecular subgroups {[7]; WNT, SHH, Group 3 (GRP3) and Group 4 (GRP4)}. The dataset A was not divided in these subgroups, due to a reduced number of samples. Abbreviations: NC, normal merebellum; MB, medulloblastoma; GRP3, Group 3; GRP4, Group 4.

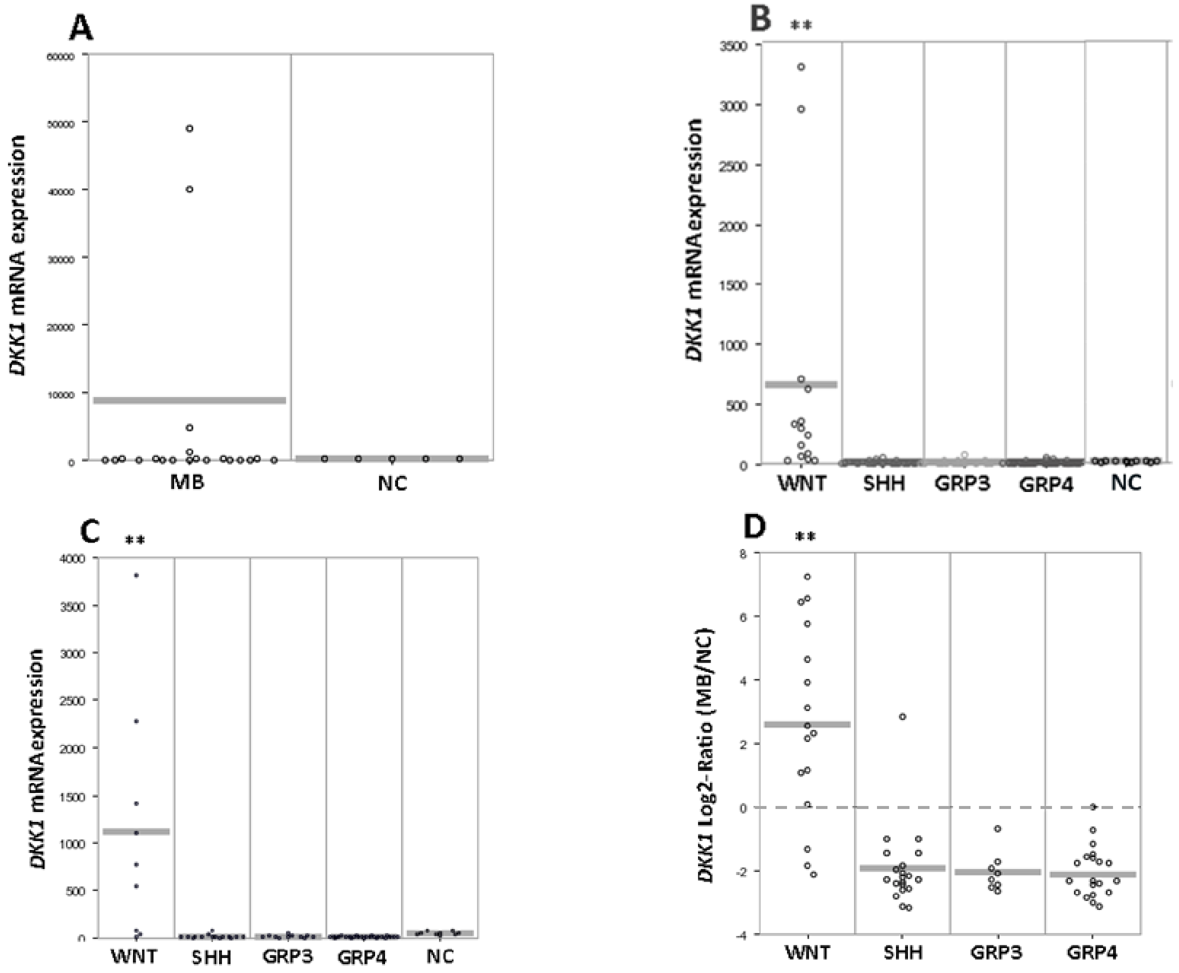

Figure S2. Dot plots of *DKK2* expression values in medulloblastoma samples (MB) and normal cerebellum (NC) from four independent datasets by gene expression analysis: (**A**) 19 MB tumors and two pools of NC (present dataset); (**B**) 188 MBs and 11 NC data [4]; (**C**) 62 MB data [2] and nine NC [20]. Differences between two groups (MB samples versus NC and WNT subgroup *versus* NC) were studied using the two-tailed Student's *t*-test. *p* values < 0.01 were considered to be statistically significant (**). The X-axis indicates the samples grouped into the four molecular subgroups {[7]; WNT, SHH, Group 3 (GRP3) and Group 4 (GRP4)}. The dataset **A** was not divided in these subgroups, due to a reduced number of samples. The dataset **D** is not present because *DKK2* probes were removed after data normalization since more than 80% values across all samples are missed. Abbreviations: NC, normal merebellum; MB, medulloblastoma; GRP3, Group 3; GRP4, Group 4.

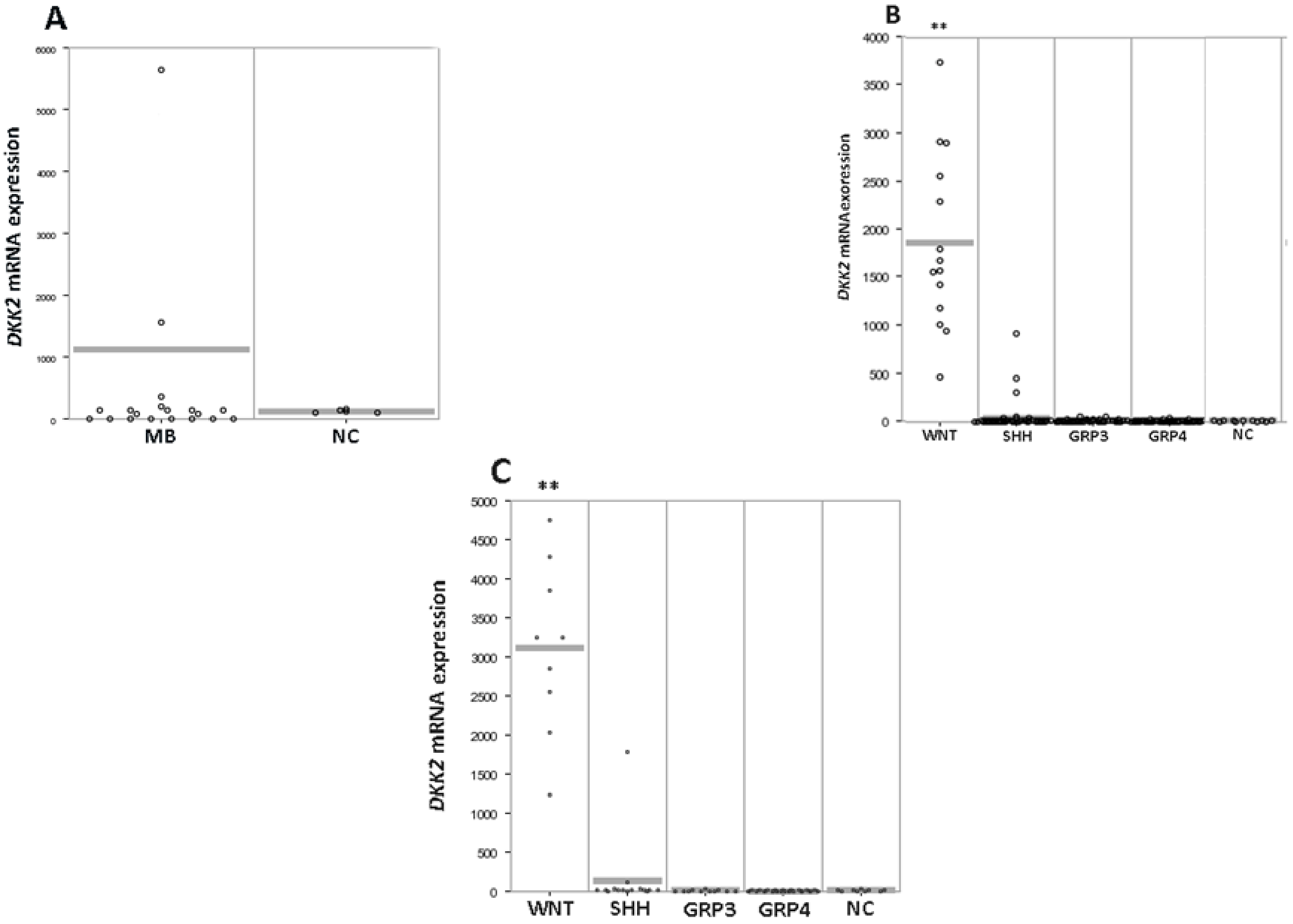

Figure S3. Dot plots of *DKK4* expression values in medulloblastoma samples (MB) and normal cerebellum (NC) from four independent datasets by gene expression analysis: (**A**) 19 MB tumors and 2 pools of NC (present dataset); (**B**) 188 MBs and 11 NC data [4]; (**C**) 62 MB data [2] and 9 NC [20]. Differences between two groups (MB samples *versus* NC and WNT subgroup versus NC) were studied using the two-tailed Student's *t*-test. *p* values < 0.01 were considered to be statistically significant (**). The X-axis indicates the samples grouped into the four molecular subgroups {[7]; WNT, SHH, Group 3 (GRP3) and Group 4 (GRP4)}. The dataset A was not divided in these subgroups, due to a reduced number of samples. The dataset D is not present because *DKK4* probes were removed after data normalization since more than 80% values across all samples are missed. Abbreviations: NC, normal merebellum; MB, medulloblastoma; GRP3, Group 3; GRP4, Group 4.

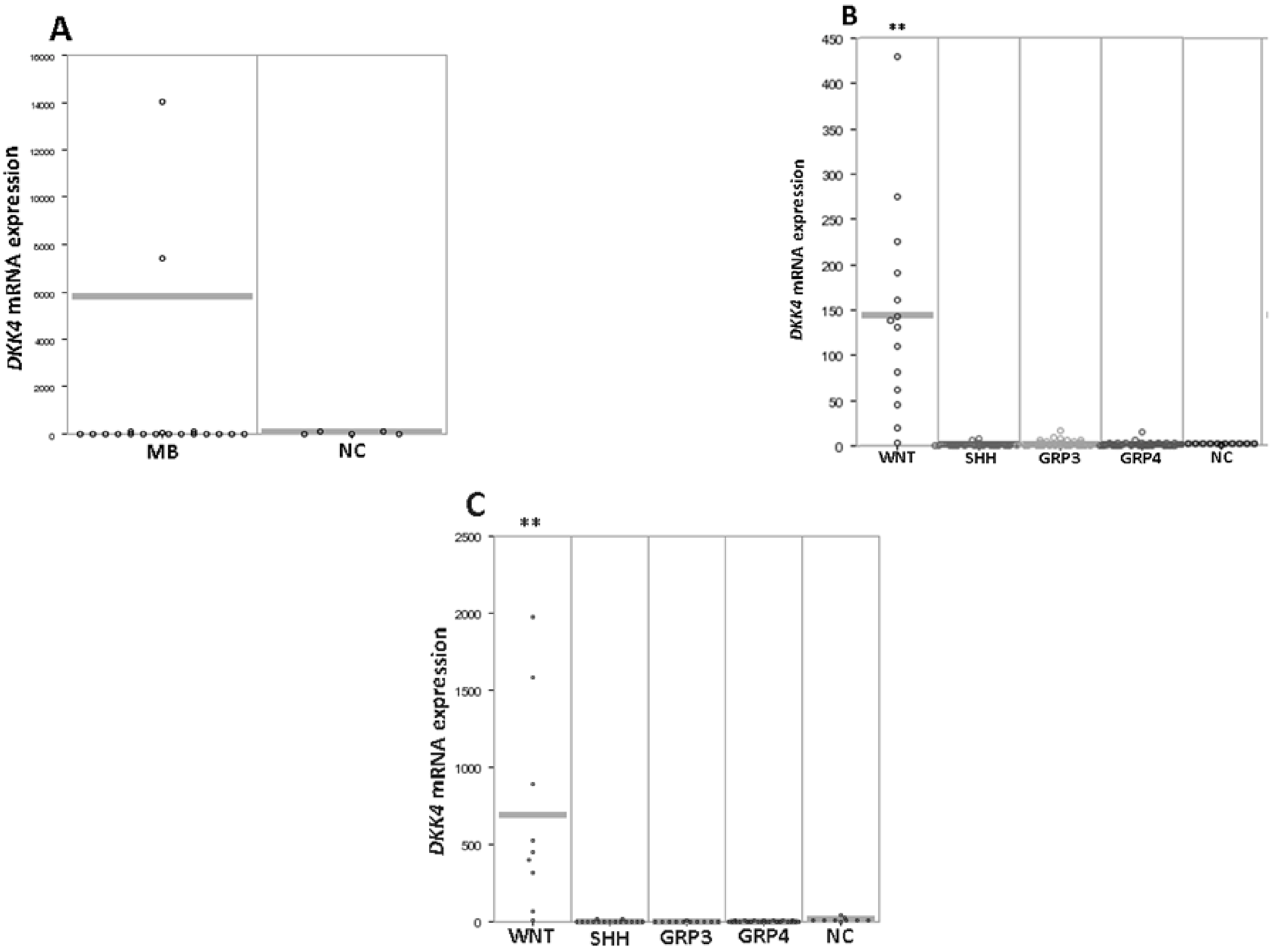

Figure S4. Representative pyrograms of the methylation level of the three *DKK3*'s promoter regions (promoter 1, 2 and 3) of three MB tumor samples.

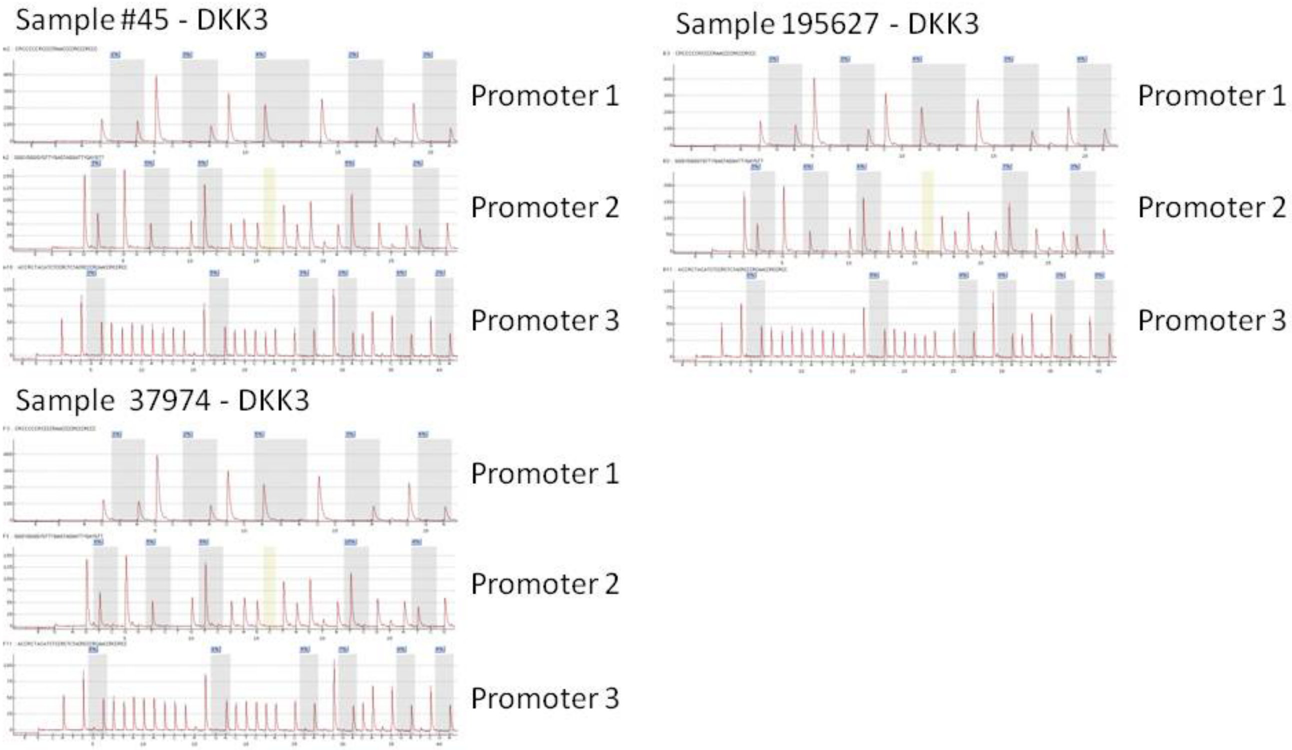

Table S1. Predictive microRNAs on DKK3 3' UTR region by 5 predictive tools. Predictive microRNAs on DKK3 3' UTR region was identified using five miRNA target prediction programs: miRWalk, Diana-microT; miRanda, miRDB and TargetScan. We selected 147 miRNAs present in at least 3 out of 5 five predictive tools; 77 out of 147 the expression values were present. The analysis was performed using the miRNA/mRNA expression data available from 25 tumors of dataset D. Statistical analysis was performed calculating the respective Pearson's rank correlation coefficient and a significant negative correlation was defined for $r2 < -0.5$. 1: miRNA present in the prediction program; 0: absent. In the column SUM is reported how many time each miRNA is present in the prediction programs.

MicroRNA	DIANAmT	miRanda	miRDB	miRWalk	Targetscan	SUM	Pearson's inverse correlation coefficient
hsa-miR-371-5p	1	1	1	1	1	5	−0.285075443
hsa-miR-488	1	1	1	1	1	5	−0.095653899
hsa-miR-450b-5p	1	1	1	1	1	5	not present
hsa-miR-579	1	1	1	1	1	5	not present
hsa-miR-657	1	1	1	1	1	5	not present
hsa-miR-891b	1	1	1	1	1	5	not present
hsa-miR-939	1	1	1	1	1	5	not present
hsa-miR-518a-5p	1	1	0	1	1	4	−0.468876521

hsa-miR-507	1	1	0	1	1	4	−0.437143413
hsa-miR-92a	1	1	0	1	1	4	−0.429416337
hsa-miR-92b	1	1	0	1	1	4	−0.405626744
hsa-miR-25	1	1	0	1	1	4	−0.356534956
hsa-miR-324-3p	1	1	0	1	1	4	−0.351934904
hsa-miR-28-5p	1	1	0	1	1	4	−0.332536363
hsa-miR-506	1	1	0	1	1	4	−0.313189581
hsa-miR-212	1	1	0	1	1	4	−0.308035243
hsa-miR-146a	1	1	0	1	1	4	−0.29605848
hsa-miR-345	1	1	0	1	1	4	−0.260334671
hsa-miR-452	1	1	0	1	1	4	−0.244987603
hsa-miR-515-5p	1	1	0	1	1	4	−0.237850331
hsa-miR-185	1	1	0	1	1	4	−0.236411384
hsa-let-7c	1	1	0	1	1	4	−0.228371113
hsa-miR-423-3p	1	1	0	1	1	4	−0.222941009
hsa-miR-132	1	1	0	1	1	4	−0.221660544
hsa-let-7f	1	1	0	1	1	4	−0.218559008
hsa-miR-199b-3p	1	1	0	1	1	4	−0.213486646
hsa-miR-453	1	1	0	1	1	4	−0.18111528
hsa-miR-122	1	1	0	1	1	4	−0.17491646
hsa-let-7d	1	1	0	1	1	4	−0.15269511
hsa-miR-105	1	1	0	1	1	4	−0.144955743
hsa-miR-199a-3p	1	1	0	1	1	4	−0.105654071
hsa-let-7g	1	1	0	1	1	4	−0.089055111
hsa-miR-362-3p	1	1	0	1	1	4	−0.08437388
hsa-miR-500	1	1	0	1	1	4	−0.082383003
hsa-miR-98	1	1	0	1	1	4	−0.082048295
hsa-miR-363	1	1	0	1	1	4	−0.053390982
hsa-miR-151-5p	1	1	0	1	1	4	−0.033511396
hsa-miR-346	1	1	0	1	1	4	−0.031454018
hsa-miR-202	1	1	0	1	1	4	−0.008615639
hsa-let-7e	1	1	0	1	1	4	0.008419887
hsa-miR-149	1	1	0	1	1	4	0.017416473
hsa-miR-138	1	1	0	1	1	4	0.062683401
hsa-miR-425	1	1	0	1	1	4	0.065098424
hsa-miR-374a	1	1	0	1	1	4	0.066932519
hsa-miR-150	1	1	0	1	1	4	0.074929518
hsa-let-7b	1	1	0	1	1	4	0.116088986
hsa-miR-19a	1	1	0	1	1	4	0.117365658
hsa-miR-330-3p	1	1	0	1	1	4	0.128661631
hsa-miR-7	1	1	0	1	1	4	0.138410833
hsa-miR-32	1	1	0	1	1	4	0.150256478
hsa-miR-141	1	1	0	1	1	4	0.188593895
hsa-miR-367	1	1	0	1	1	4	0.189292664
hsa-let-7a	1	1	0	1	1	4	0.206898241
hsa-let-7i	1	1	0	1	1	4	0.207458823
hsa-miR-134	1	1	0	1	1	4	0.221479036

hsa-miR-29c	1	1	0	1	1	4	0.24090292
hsa-miR-19b	1	1	0	1	1	4	0.247703769
hsa-miR-582-3p	1	1	0	1	1	4	0.249525388
hsa-miR-29a	1	1	0	1	1	4	0.255231011
hsa-miR-337-5p	1	1	0	1	1	4	0.301744803
hsa-miR-124	1	1	0	1	1	4	0.349288691
hsa-miR-379	1	1	0	1	1	4	0.349540931
hsa-miR-432	1	1	0	1	1	4	0.381912725
hsa-miR-377	1	1	0	1	1	4	0.411891362
hsa-miR-495	1	1	0	1	1	4	0.415839972
hsa-miR-329	1	1	0	1	1	4	0.431740287
hsa-miR-380	1	1	0	1	1	4	0.444034145
hsa-miR-544	1	1	0	1	1	4	0.481662953
hsa-miR-29b	1	1	0	1	1	4	0.548662542
hsa-miR-513a-3p	0	1	1	1	1	4	not present
hsa-miR-1205	0	1	1	1	1	4	not present
hsa-miR-1244	0	1	1	1	1	4	not present
hsa-miR-1278	0	1	1	1	1	4	not present
hsa-miR-1290	0	1	1	1	1	4	not present
hsa-miR-129-5p	1	1	0	1	1	4	not present
hsa-miR-146b-5p	1	1	0	1	1	4	not present
hsa-miR-200a	1	1	0	1	1	4	not present
hsa-miR-300	1	1	0	1	1	4	not present
hsa-miR-455-3p	1	1	0	1	1	4	not present
hsa-miR-527	1	1	0	1	1	4	not present
hsa-miR-532-5p	1	1	0	1	1	4	not present
hsa-miR-548b-3p	1	1	0	1	1	4	not present
hsa-miR-548c-3p	1	1	0	1	1	4	not present
hsa-miR-557	1	1	0	1	1	4	not present
hsa-miR-563	1	1	0	1	1	4	not present
hsa-miR-570	1	1	0	1	1	4	not present
hsa-miR-573	1	1	0	1	1	4	not present
hsa-miR-574-3p	1	1	0	1	1	4	not present
hsa-miR-576-3p	1	1	0	1	1	4	not present
hsa-miR-580	1	1	0	1	1	4	not present
hsa-miR-583	1	1	0	1	1	4	not present
hsa-miR-587	1	1	0	1	1	4	not present
hsa-miR-589	1	1	0	1	1	4	not present
hsa-miR-593	1	1	0	1	1	4	not present
hsa-miR-605	1	1	0	1	1	4	not present
hsa-miR-608	1	1	0	1	1	4	not present
hsa-miR-626	1	1	0	1	1	4	not present
hsa-miR-632	1	1	0	1	1	4	not present
hsa-miR-635	1	1	0	1	1	4	not present
hsa-miR-637	1	1	0	1	1	4	not present
hsa-miR-646	1	1	0	1	1	4	not present
hsa-miR-649	1	1	0	1	1	4	not present

hsa-miR-655	1	1	0	1	1	4	not present
hsa-miR-661	1	1	0	1	1	4	not present
hsa-miR-708	1	1	0	1	1	4	not present
hsa-miR-874	1	1	0	1	1	4	not present
hsa-miR-875-5p	1	1	0	1	1	4	not present
hsa-miR-877	1	1	0	1	1	4	not present
hsa-miR-888	1	1	0	1	1	4	not present
hsa-miR-892a	1	1	0	1	1	4	not present
hsa-miR-920	1	1	0	1	1	4	not present
hsa-miR-942	1	1	0	1	1	4	not present
hsa-miR-146b-3p	0	1	0	1	1	3	−0.496128921
hsa-miR-499-3p	1	1	0	1	0	3	−0.477101216
hsa-miR-186	1	1	0	1	0	3	−0.453005253
hsa-miR-125b	1	0	0	1	1	3	−0.279894668
hsa-miR-502-3p	1	1	0	1	0	3	−0.262375458
hsa-miR-342-5p	1	1	0	1	0	3	−0.158341133
hsa-miR-452*	1	1	0	1	0	3	−0.146790971
hsa-miR-501-3p	1	1	0	1	0	3	−0.095258351
hsa-miR-125a-5p	1	0	0	1	1	3	−0.034417242
hsa-miR-198	0	1	0	1	1	3	0.072910084
hsa-miR-490-5p	1	1	0	1	0	3	0.437364645
hsa-miR-432*	1	1	0	1	0	3	0.464946699
hsa-miR-380*	1	1	0	1	0	3	0.508346057
hsa-miR-1275	0	1	0	1	1	3	not present
hsa-let-7b*	1	1	0	1	0	3	not present
hsa-let-7c*	1	1	0	1	0	3	not present
hsa-miR-1184	0	1	0	1	1	3	not present
hsa-miR-1228	0	1	0	1	1	3	not present
hsa-miR-1252	0	1	0	1	1	3	not present
hsa-miR-1254	0	1	0	1	1	3	not present
hsa-miR-1270	0	1	0	1	1	3	not present
hsa-miR-1287	0	1	0	1	1	3	not present
hsa-miR-1303	0	1	0	1	1	3	not present
hsa-miR-146a*	1	1	0	1	0	3	not present
hsa-miR-516b	1	1	0	1	0	3	not present
hsa-miR-556-3p	1	1	0	1	0	3	not present
hsa-miR-561	1	1	0	1	0	3	not present
hsa-miR-589*	1	1	0	1	0	3	not present
hsa-miR-590-5p	1	1	0	1	0	3	not present
hsa-miR-593*	1	1	0	1	0	3	not present
hsa-miR-596	1	1	0	1	0	3	not present
hsa-miR-603	1	1	0	1	0	3	not present
hsa-miR-664	0	1	0	1	1	3	not present
hsa-miR-93*	1	1	0	1	0	3	not present
hsa-miR-936	1	1	0	1	0	3	not present

Table S2. Pyrosequencing analysis and qPCR validation of MB samples. Pyrosequencing analysis was performed on 32 MB tumor samples and 5 MB cell lines for DKK3 (1, 2 and 3 promoter). The results are shown as percentage of methylation of each site. DKK3 expression values by qPCR are reported for MB samples and MB cell lines in common with pyrosequencing analysis. DKK3 expression values relative to NC, lower than 0.5 were defined down-regulated.

DKK3 Methylation results (%)				
ID Sample	Promoter 1	Promoter 2	Promoter 3	qPCR
37472	*	4.08	*	n.d.
37974	3.32	4.29	6.62	n.d.
153342	2.18	3.15	5.78	0.1678
154904	3.04	4.83	5.10	0.2978
181900	2.22	3.80	4.13	0.4173
182059	2.92	4.69	5.12	0.0480
188181	1.32	4.07	2.18	0.0635
193263	0.79	4.04	4.33	1.0603
195589	3.44	6.02	3.39	n.d.
195615	1.92	3.99	4.88	0.1774
195627	3.16	4.20	2.45	n.d.
196684	3.38	4.08	3.85	n.d.
#29	5.29	5.41	11.64	n.d.
#32	2.94	6.89	5.47	n.d.
#33	6.51	6.86	7.12	n.d.
#45	2.64	5.59	3.33	n.d.
104-539B	3.10	11.24	10.48	n.d.
104-774	3.05	4.79	4.24	n.d.
160-I-00	3.34	6.57	*	n.d.
180-I-00	5.36	*	5.07	n.d.
2991-03	1.79	5.89	2.96	n.d.
3023-I-03	3.37	6.97	3.23	n.d.
D283	3.31	3.04	4.69	2.2068
D341	2.69	2.12	6.62	0.0001
D425	3.43	4.20	5.01	0.0012
D458	3.57	6.16	6.81	0.0001
DAOY	2.06	1.69	4.73	0.7596
*D.	3.83	5.76	6.72	n.d.
I05 - 2491	11.98	25.75	5.12	n.d.
MB005	1.98	6.41	3.09	n.d.
MB007	5.06	*	*	0.0003
MB008	7.78	*	22.00	2.0735
MB009	6.02	3.89	23.63	0.4276
MB011	2.77	6.02	3.50	n.d.
S.	*	4.10	*	n.d.

*: failed; n.d.: not done.

Table S3. Summary of clinical information of patients enrolled in the study. Histological subtype was defined according to the current WHO [35]; Metastasis stage was defined following the Chang Classification [36]. Array-CGH data were performed by Coco *et al.* [31]. Gene Expression data were uploaded in GEO (GSE39182).

**Sex**	**Age**	**Metastasis**	**Surgical tumor Residual**	**Event**	**Death**	**Follow-up**	**Outcome**	**Pyrosequencing**	**qPCR analysis**	**array-CGH**	**Gene Expression data**
F = Female<br>M = Male	months	0 = M0<br>1 = M1<br>2 = M2<br>3 = M3<br>4 =M4	0 = mikroscop. no tumo<br>1 = tumor residual <1.5 cm^2<br>2 = tumor residual >1.5 cm^2	0 = no<br>1 = yes	0 = no<br>1 = yes	months	ANED: Alive, no evidence disease<br>AWD: Alive with disease DOD Dead with disease	0 = no<br>1 = yes	0 = no<br>1 = yes	0 = no<br>1 = yes	0 = no<br>1 = yes
M	95	0	1	1	1	30	DOD	1	0	0	0
F	97	0	0	0	0	95	ANED	1	0	0	0
F	60	0	0	1	0	89	ANED	0	1	0	0
M	29	0	2	1	1	9	DOD	0	1	0	0
M	20	0	0	0	0	83	ANED	0	1	0	0
F	17	0	0	0	0	67	ANED	0	1	0	0
M	92	0	0	0	0	1329	ANED	0	1	0	0
M	20	0	0	0	0	78	ANED	0	1	0	0
M	28	0	0	1	1	18	DOD	0	1	0	0
M	109	0	0	1	1	5	DOD	0	1	0	0
M	69	0	0	1	1	14	DOD	0	1	0	0
F	31	0	2	1	1	12	DOD	0	1	0	0
M	142	0	0	1	1	46	DOD	0	1	0	0
M	48	3	0	1	1	6	DOD	0	1	0	0
M	24	3	0	1	1	7	DOD	1	0	0	0
F	24	0	0	1	1	n.d.	DOD	0	1	0	0
M	90	0	0	0	0	37	ANED	1	0	0	0
F	56	0	0	0	0	40	ANED	1	0	0	0
F	140	0	0	0	0	117	ANED	0	1	0	0

F	130	2	0	0	0	85	ANED	1	0	0	0
F	143	0	0	1	1	23	DOD	1	0	0	0
F	50	3	0	1	1	18	DOD	1	0	0	0
M	37	2	2	1	1	5	DOD	1	0	0	0
M	76	3	1	1	1	10	DOD	1	0	0	0
M	5	0	2	0	0	14	DOD	1	0	0	0
M	176	2	0	1	1	21	DOD	1	0	0	0
M	129	0	0	0	0	52	ANED	0	1	0	1
M	40	0	1	0	0	66	ANED	0	1	0	1
M	66	0	0	1	0	94	ANED	1	0	1	0
M	90	3	0	0	0	87	ANED	1	0	1	0
M	98	0	0	1	1	22	DOD	1	1	1	0
M	39	0	0	0	0	155	ANED	1	1	1	0
M	142	0	0	0	0	137	ANED	1	0	1	0
F	135	0	1	0	0	135	ANED	1	0	1	0
M	82	0	1	1	1	69	DOD	1	0	1	0
M	100	1	0	1	1	88	DOD	1	0	1	0
M	104	3	2	1	1	n.d.	DOD	1	0	1	0
F	59	0	1	0	0	76	ANED	1	0	1	0
F	45	0	0	1	1	74	DOD	1	1	1	1
M	51	0	1	1	1	32	DOD	1	1	1	1
M	49	0	1	1	1	20	DOD	1	1	1	1
M	129	0	0	1	1	19	DOD	1	1	1	1
M	141	2	0	1	1	28	DOD	1	1	1	1
M	39	0	0	0	0	155	ANED	1	0	1	1
F	49	0	0	1	0	79	ANED	0	1	1	1
M	50	0	0	0	0	69	ANED	0	1	1	1
M	50	0	0	0	0	75	ANED	0	1	1	1
M	63	2	2	1	0	97	ANED	0	1	1	1
F	83	0	0	0	0	115	ANED	0	1	1	1
F	114	0	0	0	0	134	ANED	0	1	1	1

M	71	0	0	1	0	87	AWD	1	1	1	1
M	155	0	0	0	0	169	ANED	1	1	1	1
F	117	0	0	0	0	127	ANED	1	1	1	1
M	27	0	0	1	0	31	AWD	0	1	1	1
F	129	0	0	0	0	137	ANED	1	0	1	1

n.d.: not done.

Table S4. Oligonucleotides primers and PCR conditions used in the methylation study. Pyrosequencing and in Chromatin ImmunoPrecipitation (ChIP) was performed for DKK3 gene (1, 2 and 3 promoter) using specific primers.

Pyrosequencing	Sense Primer 5'-3'	TA (°C)	Amplicon lenght (bp)
DKK3 Pro 1	Forward: CCCACCAAACTCCCTCTC<br>Reverse: BIO-AGGGGTAGGTAGTGAAGGAGATG<br>Sequencing: CACCAAACTCCCTCTC	56.5	188
DKK3 Pro 2	Forward: GGGAGGGGAYGATTTTGTTGA<br>Reverse:BIO-TTCCACCTCAAACCTCTCTCA<br>Sequencing: TTTTTTGGTGGATGTG	56	141
DKK3 Pro 3	Forward: CCRCCRCCAACAACAAAC<br>Reverse: BIO-GGYGGGTGYGGGAAAGAGG<br>Sequencing: CAACAAAATAACCCCAA	62	196
**CHIP**	**Sense Primer 5'-3'**	**TA (°C)**	**PCR Cycles**
DKK3 Pro 1	Forward: CCACTTCCACCTCAAGCCTCTCTC<br>Reverse: GAGCTCAGCCTCTCTTGGTGGATG	60	40
DKK3 Pro 2	Forward: AAGCCTGGAGGCCAGAGAA<br>Reverse: AGCGACCAGCTCTACCGAAG	60	40
DKK3 Pro 3	Forward: GCTCAGCTTGTGCTCGAGGATG<br>Reverse: AGACGGGAGGAAACCGAGG	60	40

PRO, promoter; TA (°C), Temperature of Annealing; UM, UnMethylated; M, Methylated.

© 2013 by the authors; licensee MDPI, Basel, Switzerland. This article is an open access article distributed under the terms and conditions of the Creative Commons Attribution license (http://creativecommons.org/licenses/by/3.0/).
